# Stereotactic body radiotherapy with periprostatic hydrogel spacer for localized prostate cancer: toxicity profile and early oncologic outcomes

**DOI:** 10.1186/s13014-019-1346-5

**Published:** 2019-08-02

**Authors:** Mark E. Hwang, Mark Mayeda, Maria Liz, Brenda Goode-Marshall, Lissette Gonzalez, Carl D. Elliston, Catherine S. Spina, Oscar A. Padilla, Sven Wenske, Israel Deutsch

**Affiliations:** 1 Department of Radiation Oncology, Columbia University Medical Center, New York, 10032 USA; 2 Department of Urology, Columbia University Medical Center, New York, 10032 USA

**Keywords:** Prostate cancer, Stereotactic body radiotherapy, Rectal toxicity, SpaceOAR hydrogel, Dosimetry

## Abstract

**Background:**

Multiple phase I-II clinical trials have reported on the efficacy and safety of prostate stereotactic body radiotherapy (SBRT) for the treatment of prostate cancer. However, few have reported outcomes for prostate SBRT using periprostatic hydrogel spacer (SpaceOAR; Augmenix). Herein, we report safety and efficacy outcomes from our institutional prostate SBRT experience with SpaceOAR placement.

**Methods:**

Fifty men with low- or intermediate-risk prostate cancer treated at a single institution with linear accelerator-based SBRT to 3625 cGy in 5 fractions, with or without androgen deprivation therapy (ADT) were included. All patients underwent SpaceOAR and fiducial marker placement followed by pre-treatment MRI. Toxicity assessments were conducted at least weekly while on treatment, 1 month after treatment, and every follow-up visit thereafter. Post-treatment PSA measurements were obtained 4 months after SBRT, followed by every 3–6 months thereafter. Acute toxicity was documented per RTOG criteria.

**Results:**

Median follow up time was 20 (range 4–44) months. Median PSA at time of diagnosis was 7.4 (2.7–19.5) ng/ml. Eighteen men received 6 months of ADT for unfavorable intermediate risk disease. No PSA failures were recorded. Median PSA was 0.9 ng/mL at 20 months; 0.08 and 1.32 ng/mL in men who did and did not receive ADT, respectively. Mean prostate-rectum separation achieved with SpaceOAR was 9.6 ± 4 mm at the prostate midgland.

No grade ≥ 3 GU or GI toxicity was recorded. During treatment, 30% of men developed new grade 2 GU toxicity (urgency or dysuria). These symptoms were present in 30% of men at 1 month and in 12% of men at 1 year post-treatment. During treatment, GI toxicity was limited to grade 1 symptoms (16%), although 4% of men developed grade 2 symptoms during the first 4 weeks after SBRT. All GI symptoms were resolving by the 1 month post-treatment assessment and no acute or late rectal toxicity was reported > 1 month after treatment.

**Conclusions:**

Periprostatic hydrogel placement followed by prostate SBRT resulted in minimal GI toxicity, and favorable early oncologic outcomes. These results indicate that SBRT with periprostatic spacer is a well-tolerated, safe, and convenient treatment option for localized prostate cancer.

**Electronic supplementary material:**

The online version of this article (10.1186/s13014-019-1346-5) contains supplementary material, which is available to authorized users.

## Introduction

The proportion of men with localized prostate cancer treated with stereotactic body radiotherapy (SBRT) has risen with an accumulation of oncologic and toxicity outcomes data that compare favorably with those for conventionally-fractionated radiotherapy [1–3]. However, a recent report suggests that less than 10% of men with low- and intermediate-risk prostate cancer are treated with SBRT [4].

For the first time, the 2018 National Comprehensive Cancer Network (NCCN) Guidelines includes five-fraction prostate SBRT regimens (36.25, 37 or 40 Gy) for men with very low- to favorable intermediate-risk prostate cancer as a treatment option. This recommendation is based on multiple single-institution retrospective series [5–9], phase II analyses [3, 10], and at least two prospective multicenter studies [11–13] that showed 7-year biochemical progression-free survival of 95.5% for patients with low-risk, 91.4% for favorable intermediate-risk and 85.1% for unfavorable intermediate-risk prostate cancer following 4- or 5-fraction SBRT techniques.

Despite the successful implementation of radical radiotherapy of 36 Gy in six fractions for prostate cancer over 50 years ago [14], there is a paucity of prospective data with long-term follow up reporting toxicity outcomes, thus limiting widespread adoption of prostate SBRT. A retrospective series by Katz et al. represents the prostate SBRT cohort with the longest follow-up, reporting 10-year biochemical progression-free survival of 93% in low risk prostate cancer following 35–36.25 cGy in five fractions [15].

The benefits of shorter treatment time and lower cost of prostate SBRT are weighed against the concern for higher rates of genitourinary (GU) and gastrointestinal (GI) toxicities, compared with conventionally-fractionated radiotherapy [16–18]. One approach implemented to reduce the rectal wall dose and thus minimize GI toxicity is to temporarily enlarge the perirectal space using a dissolvable, biocompatible hydrogel. The Augmenix SpaceOAR hydrogel received FDA approval following publication of a phase III clinical trial in 2014 that showed a statistically significant reduction in acute rectal pain in men treated with conventionally fractionated prostate radiotherapy, and improvement in late grade 1 toxicity (5.6% v 2%) 3–15 months after treatment [19]. Another study showed that with additional follow up, late grade 1 rectal toxicity at 3 years was still significantly lower in the SpaceOAR arm compared to treatment without SpaceOAR (42% v 17%, *p* = 0.04) [20]. We hypothesized that the toxicity improvement attributed to increased perirectal spacing is likely to be equally, if not more, pronounced for men treated with higher dose per fraction SBRT.

While the use of the hydrogel spacer has increased with SBRT practice [10], long-term follow-up is limited by its recent FDA approval. Only a small number of studies has demonstrated improved dosimetry and projected improved cost-effectiveness for SBRT in the setting of hydrogel use [21–23]. Herein we report our toxicity and early oncologic outcomes in a cohort of 50 men receiving prostate SBRT following SpaceOAR hydrogel placement for low and intermediate risk prostate cancer.

## Methods

We conducted a single-institution, retrospective chart review of patients with newly diagnosed low- and intermediate-risk prostate cancer between 2015 and 2018. Risk groups were defined using D’Amico and Zumsteg criteria [24]. We identified 50 consecutively-treated patients (Table 1) who received SpaceOAR hydrogel placement followed by prostate SBRT to 3625 cGy in 5 daily fractions delivered twice a week.

**Table 1:** Patient baseline characteristics

Age ± stdev, y	69±7.5
Range, y	50-82
PSA ± stdev, ng/mL	7.4±3.2
Range, ng/mL	2.7-19.5
Gleason score 6	8 (16%)
Gleason score 7	42 (84%)
Primary GS 4	19 (38%)
Cores involved ± stdev (#)	4.1±2.4
Range	1-11
Cores involved ± stdev (%)	32±18
Range	8-80
Clinical T stage:	
cT1c, %	86
cT2a, %	12
cT2b, %	2
NCCN Risk Stage	
Low	8 (16%)
Intermediate, favorable	16 (32%)
Intermediate, unfavorable	26 (52%)
AUA score ± stdev	9±7
Range	0-25
SHIM score ± stdev	13±8.5
Range	0-26
Prostate volume ± stdev (cc)	63±28
Range	30-109

Eighteen of 26 (69%) men with unfavorable intermediate risk prostate cancer received androgen-deprivation therapy (ADT). ADT was initiated with bicalutamide 50 mg daily for 28 days and the first leuprolide depot injection approximately 2 weeks after starting bicalutamide. Neoadjuvant ADT lasted approximately 2 months before radiotherapy commenced, with the total duration of ADT lasting 6 months. All patients initiated therapy (radiotherapy or ADT) within 6 months of a biopsy-proven diagnosis of prostate cancer.

Patients underwent simultaneous periprostatic SpaceOAR hydrogel and MRI-compatible Cybermark gold fiducial prostate marker (CIVCO Medical Instruments Co., Inc. Kalona, IA) placement prior to starting radiotherapy. The median time from hydrogel placement to first SBRT treatment was 29 (range 14–56) days.

CT simulation was performed following rectal Fleet enema and Foley catheter placement to facilitate urethra delineation. Radiotherapy treatment was planned on the CT simulation scan fused with post-hydrogel T2-weighted prostate MRI to facilitate hydrogel delineation. The median time from hydrogel placement to prostate MRI was 15 (range 1–37) days. The clinical target volume (CTV) comprised the prostate and proximal one-third of the seminal vesicles. The planning target volume (PTV) was defined as a 3 mm expansion from the CTV in all dimensions as described in Hannan et al.[25]. Organ-at-risk (OAR) dosimetry parameters were followed as defined in RTOG 0938.

SBRT was delivered using a Varian Truebeam linear accelerator twice-weekly with Eclipse-based planning (Varian Medical Systems, Palo Alto, Ca). The mean beam-on treatment time was 262 ± 38 (range: 199–363) seconds per fraction. The median duration of treatment from first to final SBRT treatment was 15 (range 13–16) days.

PSA was obtained prior to treatment, 4 months after treatment, and every 3–6 months thereafter. In the 18 men treated with ADT for unfavorable intermediate risk prostate cancer, PSA was measured monthly until a nadir was achieved before initiating hydrogel placement and SBRT treatment. ADT was completed at the latest 4 months after SBRT. Biochemical PSA failure was defined by the Phoenix definition (i.e. nadir+ 2 ng/mL).

Acute and late toxicities were physician-assessed and recorded per RTOG grading criteria. CTCAE toxicity is also reported in Additional file 1. Toxicities were evaluated at 1 and 4 months after SBRT, and at each subsequent follow-up every 3–6 months thereafter. Patient self-reported urinary and sexual function metrics, in the form of the seven-question American Urological Association (AUA) Symptom Score (International Prostate Symptom Score, or IPSS) and the five-question Sexual Health Inventory for Men (SHIM) questionnaires were collected at baseline. Men with an AUA score exceeding 18 or prior TURBT were recommended to undergo conventionally fractionated radiotherapy rather than SBRT.

## Results

Between 2015 to 2018, 50 men with prostate cancer received SBRT following hydrogel placement. Patient characteristics are described in Table 1. Eight men had low-, 16 favorable intermediate-, and 26 unfavorable intermediate-risk prostate cancer. All men with unfavorable intermediate risk disease were recommended to be treated with a 6 month course of ADT; eight declined ADT, choosing to pursue prostate SBRT alone. ADT was completed in all men when the first post-radiotherapy PSA was obtained 4 months after treatment.

### Dosimetry

**Fig. 1:**
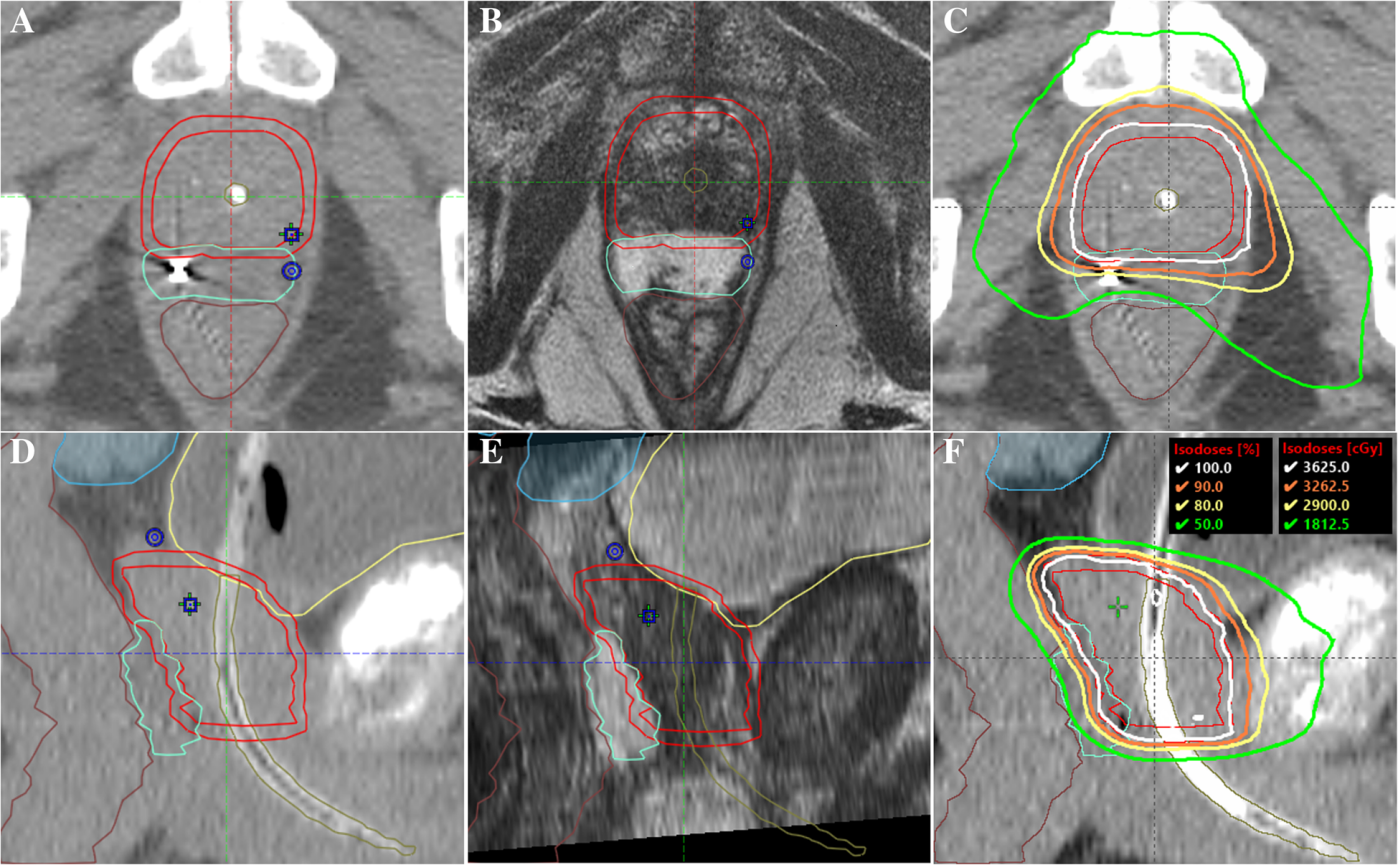
Mid-gland axial section of prostate SBRT contours showing CTV (red), PTV (red), hydrogel spacer (teal) and rectum (brown) on CT simulation scan (**a**) and fused T2-weighted MRI (**b**), and treatment plan (**c**). Corresponding sagittal views are also shown (**d**-**f**)

Figure 1 demonstrates target and OAR contours with CT-MRI fusion, and resulting treatment plan. Rectum and bladder dose constraints outlined in RTOG 0938 were met in 100% of men, generally by a wide margin (Table 2). Urethral dose constraints were met in 96% of men.

**Table 2:** PTV and OAR dosimetry with hydrogel spacer

Organ	RTOG 0938	Achieved Dose		
	Parameters (Gy)	(mean±stdev)		
PTV				
Maximum point dose (1cc)	≤ 38.78	38.1	±	0.5
Minimum dose received by 95% of PTV	≥ 36.25	36.3	±	0.4
Rectum				
Maximum point dose (1cc)	≤ 38.06	33	±	3.4
Less than 3 cc	< 34.40	29.5	±	4.0
90% rectum	≤ 32.63	25.0	±	4.4
80% rectum	≤ 29.00	21.1	±	4.7
50% rectum	≤ 18.13	13.5	±	4.7
Bladder				
Maximum point dose (1cc)	≤ 38.06	37	±	0.4
90% bladder	≤ 32.63	22.9	±	5.7
50% bladder	≤ 18.13	4.4	±	3.5
Urethra	≤ 38.78	38	±	0.4

The PTV mean and standard deviation measured 103 ± 43 cc. The perirectal distance separating the prostate CTV from the anterior rectal wall following hydrogel placement was 9.6 ± 4 mm measured at the prostate midgland, at midline. The perirectal separation measured at this location has previously been shown to be predictive of rectum dosimetry [26].

### PSA response

**Fig. 2:**
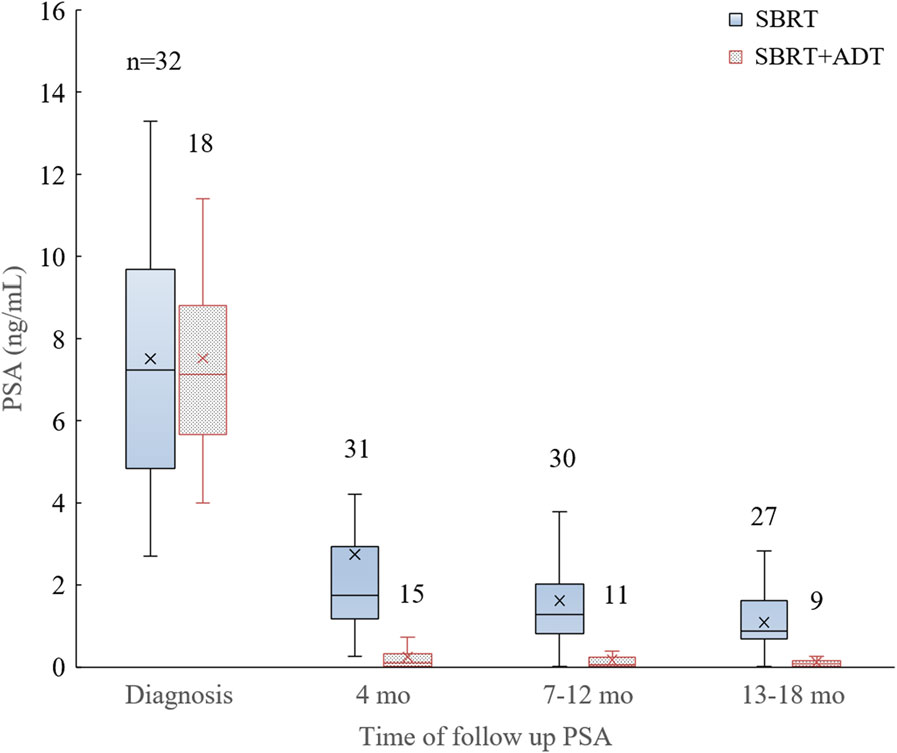
Box plot of PSA kinetics in men receiving SBRT monotherapy and SBRT+ADT. Number of evaluable men (n) denoted above each box

At a median follow up of 20 (range: 4–44) months, PSA was significantly reduced in all patients compared to pre-treatment PSA, with a median PSA of 0.9 ± 2 ng/mL (25-75th percentile, 0.22–1.37 ng/mL). No biochemical PSA failures were recorded. One patient death was recorded unrelated to prostate cancer. PSA kinetics are shown in Fig. 2.

Of the 32 men who did not receive ADT, PSA nadir was achieved in six men at 26 ± 2.5 mo (mean, stdev, range 22–28 mo) months post-SBRT. The remainder had not achieved a PSA nadir at time of last follow-up.

A PSA bounce, defined as PSA rise > 0.2 ng/mL from the nadir, was observed in 10 men who did not receive ADT at 16 ± 4 mo (mean, stdev, range 10–20 mo). Two men treated with ADT experienced a bounce at 16 and 23 months. The PSA bounce magnitude was 0.9 ± 0.4 ng/mL (mean, stdev), with PSA declining to pre-bounce nadir when PSA was repeated 3–6 months later. One patient experienced two bounces at 17 and 38 months.

### Toxicity

A summary of toxicity outcomes is shown in Table 3. Overall, 30% of men developed acute grade 2 GU toxicity during radiotherapy. Symptoms completely resolved in 12% of men in the following weeks. However, 12% of men who were asymptomatic (grade 0) at the end of treatment subsequently developed grade 2 symptoms, resulting in 30% of men reporting grade 2 GU toxicity at the 1-month post treatment follow-up visit. Grade 2 GU toxicity persisted in approximately one-sixth of all patients from 4 through 18 months. No new GU toxicities were observed after 1 month, nor was a GU “symptom flare” phenomenon evident at any point post-treatment.

**Table 3:** Percent of patients with RTOG grades 0-2 gastrointestinal (GI) and genitourinary (GU) toxicity

Toxicity	Grade	*n*	Months post treatment				
			During SBRT	1	4	7-12	13-18
			*50*	*50*	*46*	*41*	*36*
GI	0		84%	88%	100%	100%	100%
	1		16%	8% (6%)^a^	0	0	0
	2		0	4% (4%)^a^	0	0	0
GU	0		38%	44%	63%	71%	75%
	1		32%	26% (4%)^a^	20%	17%	14%
	2		30%	30% (12%)^a^	17%	12%	11%

^a^Percent in parentheses at one-month post treatment represents patients that were asymptomatic during SBRT but developed toxicity during the 4 weeks after completing radiotherapy

Mild grade 1 gastrointestinal toxicity was seen in 8 patients (16%) during radiotherapy, resolving in all but 1 patient within 1 month. No medical management for changes in bowel function was necessary during radiotherapy. In the 2 weeks after SBRT, an additional 3 patients (6%) developed mild grade 1, and 2 patients (4%) developed grade 2, toxicities requiring Imodium for symptomatic relief. All GI toxicities were resolving by the 1 month post-SBRT follow-up. No new GI toxicities were reported after this time. No toxicity related to hydrogel placement was observed.

A summary of acute GI and GU toxicity rates 1 month after 5-fraction regimens to doses of 33.5–37.5 Gy, from five recent SBRT series without the hydrogel spacer, are presented in Table 4 for comparison [5, 27–31]. Compared to SBRT without hydrogel spacer, these data demonstrate that spacer placement prior to treatment offered the most favorable GI toxicity profile, with 88% of men being asymptomatic 1 month following treatment compared to 21–62%.

**Table 4:** Comparison of acute GI and GU toxicity rates 1 month following 5-fraction prostate SBRT. A perirectal spacer was not used in the earlier series shown below

Study		Current series	Madsen (IJROBP 2006)	Loblaw (Rad Oncol 2013)	Katz (BMC Urol 2010)	Chen (BMC Rad Onc 2013)	Park (BMC Rad Onc 2018)	McBride (Cancer 2011)
Dose (Gy)		36.25	33.5	35	35-36.25	35-36.25	35-36.25	36.25-37.5
Grade (GI)	*n*	*50*	*39*	*84*	*304*	*100*	*88*	*42*
0		88%	61%	23%	21%	60%	58%	62%
1		8%	26%	67%	74%	35%	36%	31%
2		4%	13%	10%	5%	5%	6%	7%
Grade (GU)								
0		44%	49%	9%	20%	29%	42%	21%
1		26%	28%	71%	75%	36%	49%	60%
2		30%	21%	19%	5%	35%	9%	19%
3		0%	2%	1%	0%	0%	0%	0%

## Discussion

Our institutional experience represents one of few prostate SBRT series reporting outcomes following pre-treatment hydrogel spacer placement in an effort to improve rectal dosimetry and decrease toxicity. Lower rates of acute rectal toxicity were observed compared with previous, similarly-fractionated SBRT reports that were performed without spacer placement (Table 4). While widespread practitioner familiarity and favorable outcomes with conventionally-fractionated prostate radiotherapy contribute to a high threshold for implementing ultra-hypofractionated regimens for prostate cancer, a growing body of evidence suggests comparability between SBRT and conventionally fractionated radiotherapy in both toxicity and biochemical response [1, 2, 4, 32].

### PSA response

Our PSA kinetics approximate those that have been reported for SBRT regimens ranging from 35 to 37.5 Gy in five fractions. Three different series treating men with predominantly low and intermediate risk disease have demonstrated PSA declines from a median of 5–7 ng/mL at baseline to 1 ng/mL, 1 year after SBRT [29, 31, 33]. Follow-up for an additional 3–5 years revealed a continual PSA decline, with 84% of men attaining nadir under 0.5 ng/mL and a median PSA of 0.3 ng/mL at 5 years [9]. Fuller et al. reported a median PSA nadir of 0.1 ng/mL at 42 months after SBRT of 38 Gy in four consecutive daily fractions [34]. This ablative PSA response approaches that typically observed following prostate brachytherapy, raising confidence in SBRT treatment efficacy [35]. At the time of most recent publication, PSA failure rates in these series were 2 and 4% at 36 months [31, 33], and 6–11% at 60–72 months [9, 34].

Twelve men (24%) experienced a PSA bounce in our early report. Bounce rates reported in SBRT series are variable, ranging from 12 to 61%, with median time to first bounce of 11–23 months [33, 34]. Kataria et al. reported nine biochemical failures per Phoenix definition out of 145 treated men 5 years post-treatment [9]. Interestingly, four out of 58 benign bounces (40% bounce rate) in the same series were in excess of 2 ng/mL. While the vast majority of all PSA bounces are small, it is noteworthy that approximately 30% (4 out of 13) of initial PSA rises thus meeting the Phoenix definition for failure were in fact benign.

In all patients observed to experience a PSA bounce, the kinetics were not associated with prostate cancer risk grouping or Gleason score. A PSA bounce has been correlated with improved bPFS and OS in some mature reports of brachytherapy and conventional external beam radiotherapy, although it is still too early to conclude that the same holds true for SBRT [36, 37].

An increasing number of men also receive ADT with their definitive SBRT treatment as SBRT is offered to men with unfavorable intermediate risk disease. Based on SBRT data from San Bortolo Hospital in Italy, the mean PSA curves for men who did and did not receive ADT concurrent with radiation merge at < 0.5 ng/mL after 3.5 years [33]. The PSA kinetics described herein are comparable to the data from San Bartolo Hospital at the 1-year mark (Fig. 2). However, with the paucity of reports on hormonal therapy with prostate SBRT, the prognostic significance of these features of PSA kinetics remain unclear. Multiple studies evaluating PSA kinetics after brachytherapy or external beam therapy with and without hormonal therapy demonstrate improved bPFS in men with lower nadir and shorter time to nadir [38–40]. Given the radiobiological similarity of SBRT to brachytherapy and the ablative PSA responses evident in many reports of prostate SBRT, we expect that at least some of these features will also be predictive of oncologic outcome in SBRT with the addition of ADT. The ongoing trial of ADT and SBRT versus SBRT for intermediate risk prostate cancer will shed light on this question (NCT03056638).

### Toxicity

Prostate SBRT regimens in 4 or 5 fractions are well-tolerated, particularly to doses ≤40Gy as recommended in NCCN guidelines. Toxicity rates are comparable to those seen with conventionally fractionated prostate radiotherapy. In the series by Katz et al. acute grades 1–2 urinary (GU) or rectal toxicities were observed in half to two-thirds of patients, and late grade 1–2 GU or rectal toxicities in 15 and 4% of patients, respectively. Comparisons across similar prostate SBRT series (Table 4) demonstrate that the majority of men develop some changes in urinary function up until 1 month after treatment. The proportion of men requiring medical management for acute GU toxicity i.e. grade 2, is typically under one-third. Our data are consistent with these results, with approximately one third of men requiring medical management for acute GU changes and another third experiencing only mild changes from baseline GU function during and immediately after SBRT. Thirty percent of men still have some GU symptoms and 11% require medical management for these symptoms (i.e. Grade 2 toxicity) 1–1.5 years after treatment.

Patient reported outcomes (PRO) corroborate the findings suggesting minimal late SBRT toxicity. One large multi-institutional series of 803 men reported similar urinary and sexual, but better bowel PRO scores, with SBRT to total doses of ≤40Gy compared with conventionally fractionated radiotherapy 2 years after treatment [41]. Sixty-five percent of men treated with SBRT reported no minimally detectable difference (MDD) in any of urinary, sexual and bowel PRO domains compared with pre-radiotherapy baseline, versus only 40% of men treated with conventionally fractionated radiotherapy. In the same series, only 11% of men reported bowel MDD following SBRT, compared with 30% following conventionally fractionated radiotherapy. Another 2-year report documented better urinary symptoms with SBRT of 35–40 Gy in 5 fractions, on non-consecutive days, than with moderately hypofractionated radiotherapy of 51.6–70.2 Gy in 12–26 daily treatments (MDD 14% v 33%) [42].

With the excellent rectal dosimetry afforded by the perirectal spacer (Fig. 1, Table 2), already low acute GI toxicity rates previously reported declined even further. In our cohort, early GI toxicity was limited to grade 1–2 symptoms in one out of six men, with complete resolution of all GI toxicity starting 1 month post-treatment and continuing through the remainder of follow-up. In comparison, upwards of 40% of men experienced acute grade 1 or 2 GI toxicities up to 1 month after treatment in non-spacer SBRT series. It is noteworthy that a reduction of posterior CTV to PTV expansion, sometimes to as low as 0 mm to facilitate rectal sparing and optimize dosimetry [34], was unnecessary to achieve the low rectal toxicity rates we observed.

Interestingly, the phase III SpaceOAR hydrogel randomized controlled trial with conventionally fractionated prostate radiotherapy found a late (> 3 mo), but not acute (< 3 mo), rectal toxicity benefit with the hydrogel [19, 43]. This is consistent with radiobiological theory that the prolonged treatment time inherent to conventional fractionation correlates with sparing of early-responding tissue to minimize acute toxicity e.g. rectal mucosa [44]. Hence, minimal additional acute toxicity benefit was observed with the spacer in this trial. Conversely, comparing our SBRT acute rectal toxicity rates with those previously published (with the caveat that we are comparing across different series in a non-randomized setting) suggests an acute toxicity improvement using the spacer for SBRT regimens. The increased dose per fraction and condensed treatment time likely contribute to this phenomenon as well.

### Future hydrogel spacer utility

Given the favorable rectum dosimetry and acute toxicity findings in this report of SBRT with hydrogel spacer, one might expect the benefit of the spacer to be greater still for SBRT delivered at 1) higher doses and 2) larger treatment volumes (i.e. inclusion of seminal vesicles) that are current areas of investigation. With regard to higher dose per fraction, Kim et al. reported high-grade rectal toxicity requiring surgical intervention with colostomy in a subset of patients receiving prostate-only SBRT of up to 50 Gy in five fractions without spacer placement [16]. A recent cost-effectiveness analysis using a decision tree model that weighed the projected costs of spacer use against that of avoidable rectal toxicity over a ten-year period suggested a direct correlation between spacer value and fraction size [23].

The role of hypo- and ultrahypofractionated treatment regimens in high-risk prostate cancer is controversial as target volumes are expanded to include the prostate, seminal vesicles (SV), and frequently pelvic lymph nodes. The FASTR and SATURN phase II studies have evaluated pelvic nodal irradiation to 25 Gy and prostate SBRT to 40 Gy, both delivered in five fractions [45, 46]. The FASTR study that included treatment of the whole prostate plus the proximal SV to a total dose of 40 Gy was terminated early with four out of 16 men experiencing ≥ grade 3 late rectal toxicity (bleeding). In contrast, no ≥ grade 3 late rectal toxicity was observed in SATURN, where the SV were only treated to 25 Gy while the prostate received 40 Gy. Whereas, a third high-risk prostate cancer study conducted by Murthy et al. found ≤15% early and late ≤ grade 2 GI toxicity, with no ≥grade 3 GI toxicity, when treating the prostate and SV to 35–37.5 Gy and the pelvic nodes to 25 Gy in 5 fractions [47].

The late rectal toxicity results described above suggest that insufficient separation between the prostate/SV and the rectum in the absence of spacer – not the addition of pelvic nodal treatment to 25 Gy – limits safe delivery of 5-fraction radiotherapy in high-risk disease. Placement of a perirectal spacer may offer a method to improve rectal dosimetry and toxicity and should be considered when designing future clinical trials evaluating the safety and efficacy of dose escalated SBRT or expansion of the PTV to include seminal vesicles.

## Conclusions

SBRT for low and intermediate risk prostate cancer is safe, with lower rates of acute rectal toxicity using a hydrogel spacer compared with previously published series reported without the use of a perirectal spacer. These data suggest that there may be significant potential for further toxicity reduction using the spacer for larger SBRT volumes and higher doses currently being evaluated for high risk prostate cancer.

## Additional file

Additional file 1: Percent of patients with CTCAE v.4 grades 0–2 rectal (GI) and genitourinary (GU) toxicity. (DOCX 26 kb)

## Data Availability

The datasets used and/or analyzed during the current study are available from the corresponding author on reasonable request.

## Abbreviations

ADT: Androgen deprivation therapy
bPFS: Biochemical progression-free survival
PRO: Patient reported outcomes
SBRT: Stereotactic body radiotherapy
