# Supplementary material for: Stereotactic body radiotherapy with periprostatic hydrogel spacer for localized prostate cancer: toxicity profile and early oncologic outcomes
Source: Radiat Oncol. 2019 Aug 2;14:136. doi: 10.1186/s13014-019-1346-5 (PMC6679492; doi:10.1186/s13014-019-1346-5)
Supplement: Supplementary file 1 — Percent of patients with CTCAE v.4 grades 0–2 rectal (GI) and genitourinary (GU) toxicity. (DOCX 26 kb) [file 13014_2019_1346_MOESM1_ESM.docx]

|  |  |  | **Months post treatment** | | | | | |
| --- | --- | --- | --- | --- | --- | --- | --- | --- |
|  |  |  | During SBRT | 1 | 4 | 7-12 | 13-18 |  |
| Toxicity | Grade | *n* | *50* | *50* | *46* | *41* | *36* |  |
| **GI** | 0 |  | 84% | 88% | 100% | 100% | 100% |  |
|  | 1 |  | 16% | 10% | 0% | 0% | 0% |  |
|  | 2 |  | 0% | 2% | 0% | 0% | 0% |  |
|  |  |  |  |  |  |  |  |  |
| **GU** | 0 |  | 36% | 43% | 59% | 63% | 69% |  |
|  | 1 |  | 30% | 18% | 21% | 23% | 17% |  |
|  | 2 |  | 34% | 39% | 20% | 14% | 14% |  |

Additional file 1: Percent of patients with CTCAE v.4 grades 0-2 rectal (GI) and genitourinary (GU)toxicity
